# Efficacy and Safety of Bulevirtide plus Tenofovir Disoproxil Fumarate in Real-World Patients with Chronic Hepatitis B and D Co-Infection

**DOI:** 10.3390/pathogens11050517

**Published:** 2022-04-27

**Authors:** Toni Herta, Magdalena Hahn, Melanie Maier, Janett Fischer, Johannes Niemeyer, Mario Hönemann, Albrecht Böhlig, Florian Gerhardt, Aaron Schindler, Jonas Schumacher, Thomas Berg, Johannes Wiegand, Florian van Bömmel

**Affiliations:** 1 Division of Hepatology, Department of Medicine II, Leipzig University Medical Center, 04103 Leipzig, Germany; toni.herta@medizin.uni-leipzig.de (T.H.); magdalena.hahn@medizin.uni-leipzig.de (M.H.); janett.fischer@medizin.uni-leipzig.de (J.F.); johannes.niemeyer@medizin.uni-leipzig.de (J.N.); albrecht.boehlig@medizin.uni-leipzig.de (A.B.); florian.gerhardt@medizin.uni-leipzig.de (F.G.); aaron.schindler@medizin.uni-leipzig.de (A.S.); jonas.schumacher@medizin.uni-leipzig.de (J.S.); thomas.berg@medizin.uni-leipzig.de (T.B.); johannes.wiegand@medizin.uni-leipzig.de (J.W.); 2 Institute of Medical Microbiology and Virology, Leipzig University Medical Center, 04103 Leipzig, Germany; melanie.maier@medizin.uni-leipzig.de (M.M.); mario.hoenemann@medizin.uni-leipzig.de (M.H.)

**Keywords:** bulevirtide, tenofovir disoproxil fumarate, hepatitis delta, treatment response

## Abstract

Background: The hepatitis B and D virus (HBV/HDV) hepatocyte entry inhibitor bulevirtide (BLV) has been available in Europe since July 2020, after the registrational trial MYR202. Real-life data on the efficacy and safety of BLV are sparse. Methods: We have analysed the course of treatment with BLV (2 mg/day) plus tenofovir disoproxil fumarate (TDF) (245 mg/day) in patients with chronic hepatitis delta (CHD). Virologic (≥2 log reduction in HDV RNA or suppression of HDV RNA below the lower limit of detection) and biochemical (normalisation of serum ALT) treatment responses after 24 weeks were defined according to the MYR202 trial. Results: Seven patients were recruited (four with liver cirrhosis Child–Pugh A). After 24 weeks, a virologic response was observed in five of seven and a biochemical response was seen in three of six patients with elevated serum ALT at baseline. Extended treatment data > 48 weeks were available in three cases: two presented with continuous virologic and biochemical responses and in one individual an HDV-RNA breakthrough was observed. Adverse effects were not recorded. Conclusions: The first real-life data of the approved dosage of 2 mg of BLV in combination with TDF confirm the safety, tolerability, and efficacy of the registrational trial MYR202 for a treatment period of 24 weeks and beyond.

## 1. Introduction

Chronic hepatitis delta (CHD) in patients with hepatitis B virus (HBV) and hepatitis D virus (HDV) co-infection is the most devastating form of chronic viral hepatitis, with a significantly higher risk of progression to cirrhosis and hepatocellular carcinoma compared to HBV mono-infection [1,2]. CHD is sustained by HDV, a single-stranded RNA virusoid depending on HBV for replication and encapsidation [3], and it affects approximately 12–72 million individuals worldwide [4,5]. The envelope of HDV consists of the small, medium and large form of the HBV surface protein (HBsAg). The entry of HBV and HDV into hepatocytes necessitates the binding of the pre-S1 domain of the large HBsAg to the sodium-taurocholate co-transporting polypeptide (NTCP) [6,7], a bile acid transporter expressed on the basolateral plasma membrane of human hepatocytes. Several single-nucleotide polymorphisms (SNPs) in *SLC10A1*, the coding gene of NTCP, are thought to influence HBsAg binding to NTCP and, thereby, the susceptibility of hepatocytes to HBV and HDV infection [8,9,10]. Although chronic hepatitis B can be effectively controlled with nucleos(t)ide analogues, such as as tenofovir disoproxil fumarate (TDF), this approach is ineffective in the treatment of CHD [11]. Pegylated interferon alpha (PEG-IFNα), given for a duration of 48 weeks, represents a treatment option that may lead to a sustained virologic response in one out of four CHD patients [12,13,14]. However, PEG-IFNα can be associated with major side effects and is contraindicated in patients with liver cirrhosis. Thus, effective and safe therapeutic options for patients with CHD are needed. Several antiviral compounds are being tested in clinical trials. The first-in-class HBV/HDV hepatocyte entry inhibitor bulevirtide (BLV) is currently the most promising candidate and was granted conditional approval at a dose of 2 mg/day for the treatment of adult patients with CHD and compensated liver disease from the European Medicine Agency in July 2020 [15,16]. BLV is a subcutaneously injected, synthetic pre-S1 lipopeptide that competes with the pre-S1 domain of the HBsAg for binding to NTCP on the surface of hepatocytes [3,16]. The phase II trials MYR202 and MYR203 tested the safety and efficacy of BLV in different doses and combinations with or without TDF (MYR202) or PEG-IFNα (MYR203) [11,17]. The combination of BLV and TDF was shown to induce a BLV dose-dependent suppression of HDV RNA in up to 60% of patients and a normalisation of elevated ALT levels in up to 50% of patients with CHD after 24 weeks of treatment. BLV was well-tolerated, with an asymptomatic increase of serum bile acids being the most frequent adverse event [11]. An extension of treatment to 48 weeks and the combination of BLV with PEG-IFNα in the MYR203 study increased antiviral and biochemical efficacy at the price of PEG-IFNα side effects [17]. Notably, the reduction or suppression of HBsAg was only achieved in MYR203 (BLV and PEG-IFNα). All patients in MYR202 (BLV and TDF) remained HBsAg-positive, and 93–97% showed a relapse of HDV RNA after the cessation of BLV treatment. The efficacy of longer BLV therapy (with or without PEG-IFNα) is currently being assessed in the MYR204 (phase II) and the MYR301 (phase III) trials [18,19].

Real-world experiences on the use of BLV are sparse [20,21,22,23,24,25]. We are the first to report the real-world safety and efficacy of BLV at the approved dose of 2 mg/day in combination with TDF over the observation period of 24 weeks according to treatment arm I of the MYR202 trial in a cohort of CHD patients without a disease-modifying SNP in *SLC10A1*. Moreover, we report the long-term treatment response in three patients that were treated for >48 weeks.

## 2. Results

Seven patients were recruited (four males, aged 41 ± 8.1 years, five Caucasians, two Asians, four with cirrhosis Child–Pugh A). The baseline characteristics are detailed in Table 1.

The genotyping of *SLC10A1* did not reveal a disease-modifying SNP in any of the recruited patients, as shown in Table 2.

**Figure 1:**
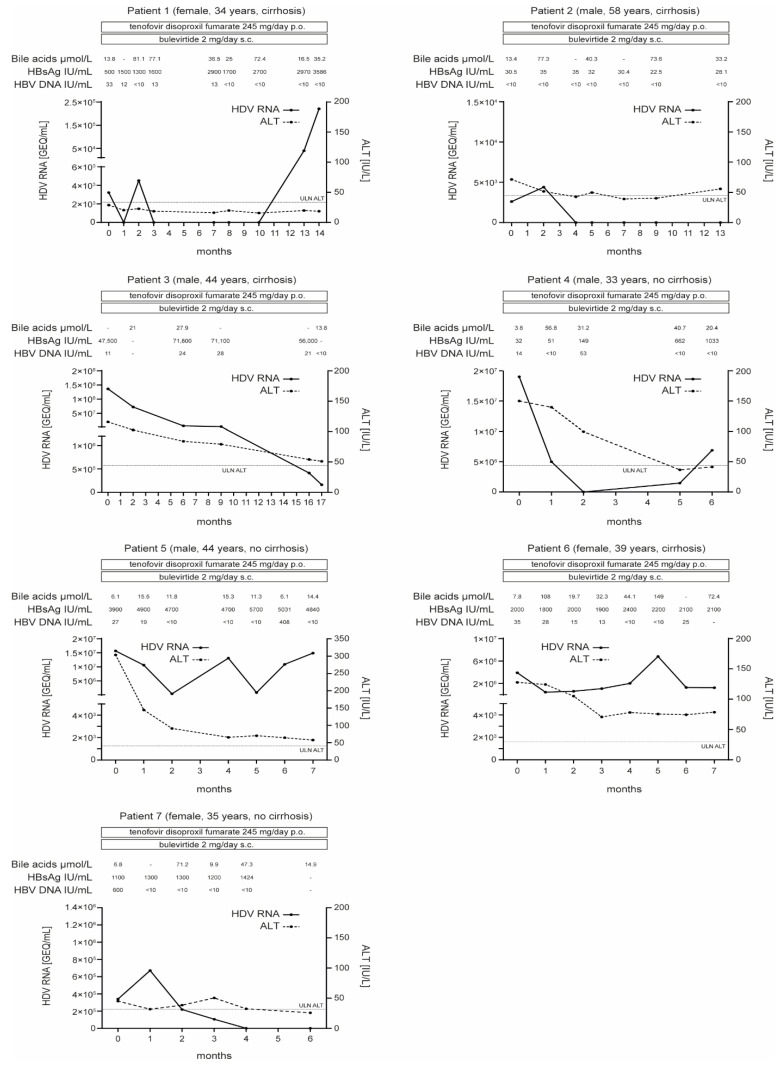
Virologic and biochemical responses to treatment. ALT, alanine aminotransferase; GEQ, genome equivalents; HBsAg, HBV surface protein; HBV, hepatitis B virus; HDV, hepatitis D virus; p.o., oral administration; s.c., subcutaneous administration; ULN, upper limit of normal; -, not avaiable.

Before treatment initiation, the median serum HDV RNA level was 3.9 × 10^6^ [range 2.6 × 10^3^ to 1.36 × 10^8^] genome equivalents (GEQ)/mL, median serum HBV DNA was 27 [range 0 to 600] IU/mL, median serum HBsAg was 1100 [range 32 to 47,500] IU/mL and the median alanine aminotransferase (ALT) level was 2.57 [range 0.82 to 6.75] above the upper limit of normal (ULN). The median time of follow-up after treatment initiation was 28 [range 24 to 68] weeks (Figure 1).

### 2.1. Response to Treatment after 24 Weeks

A virologic response was observed in five of seven patients. The median HDV RNA level at baseline was 3.4 × 10^5^ [range 2.6 × 10^3^ to 1.36 × 10^8^] GEQ/mL in virologic responders and 9.8 × 10^6^ [range 3.9 × 10^6^ to 1.57 × 10^7^] GEQ/mL in virologic non-responders. A Biochemical response was achieved in three of six patients with elevated serum ALT at baseline. All three patients were virologic responders. The remaining three patients with elevated ALT levels at baseline did not achieve a biochemical response after 24 weeks of treatment. Two of six were virologic non-responders and one showed a virologic response. One of seven patients had normal ALT levels at baseline that remained normal under treatment. This patient showed a virologic response. The combined response to treatment was achieved in three of six patients with elevated ALT at baseline.

### 2.2. Response to Treatment after >48 Weeks

Three patients received treatment for 52, 56 and 68 weeks, respectively (response after 24 weeks: 3/3 virologic response, 1/3 biochemical response and 1/3 combined response). A continuous virologic response was observed in two of three patients. ALT re-increased over the ULN in one case and continuously decreased in the other case without complete normalisation. One patient showed a breakthrough of serum HDV RNA at week 40 of therapy after initial complete suppression below the detection limit at week 12. ALT remained below the upper limit of normal during the complete course of observation in this individual. 

### 2.3. HBV Response to Treatment

HBV DNA was undetectable in six of seven patients at the end of observation. The disappearance of serum HBV DNA was also observed in one HDV virologic non-responder. No patient showed a reduction in serum HBsAg levels within the period of observation. 

### 2.4. Serum Bile Acid Levels during BLV Treatment

Five of seven patients showed a rapid ≥5-fold increase in serum bile acids compared to baseline after the initiation of treatment. For one patient, serum bile acids at baseline were not available. One virologic non-responder showed a smaller increase of 2.5-fold after the initiation of treatment. Serum bile acid levels exhibited a considerable fluctuation but remained elevated compared to baseline throughout the treatment period in six of seven patients (one virologic non-responder presented a drop to baseline after 24 weeks of treatment). The fluctuation of serum bile acid levels did not show a correlation with the fluctuation of serum HDV RNA. The elevation of serum bile acids was not accompanied by pruritus in our patients.

### 2.5. Compliance to Treatment

Compliance to treatment was reported to be high by all patients as confirmed by the elevation of serum bile acids as an indicator for NTCP target engagement. One virologic non-responder showed a lower (and non-persistent) increase in serum bile acids but a strong decline in serum ALT, which suggests adherence to treatment in this case as well.

### 2.6. Adverse Events

Clinical adverse events related to BLV were not observed. 

## 3. Discussion

We assessed the safety and efficacy of the conditionally approved BLV dose of 2 mg/day in combination with TDF, corresponding to treatment arm I of the MYR202 trial, under real-world conditions in a cohort of patients with CHD. The baseline characteristics of our cohort were similar to the respective study arm of the MYR202 trial and reflected characteristics previously reported for HDV-infected patients: all patients migrated to Germany from countries with higher HDV prevalence—in our case Eastern European countries and the former Soviet Union [5]. HDV genotype 1 (HDV-1) predominates in these countries [5,26] and was found in all of our patients. Disease-modifying SNPs in *SLC10A1* are rare in Caucasian populations [27,28] and were, thus, not found in our cohort. Non-African patients with HDV-1 are at the highest risk of cirrhosis development (>50% present with cirrhosis at the time of diagnosis) [26], which is consistent with the high rate of cirrhosis in our cohort (57% of the patients). 

Compared to the MYR202 study [11], we observed similar virologic, biochemical and combined responses after 24 weeks of treatment. BLV was well-tolerated, with an asymptomatic increase in serum bile acids being the only recorded and expected laboratory phenomenon. Thus, the virologic, biochemical and combined response rates and clinical tolerability of the MYR202 study regimen can be confirmed under real-life conditions. Previous studies suggested, that higher doses of BLV in combination with TDF might increase the virologic and combined responses but not the biochemical response to treatment [11]. Moreover, the combination of BLV (at different doses) and PEG-IFNα resulted in considerably higher virologic but similar biochemical and combined response rates in phase II [17] and under real-world conditions [21,22]. The determination of the optimal dose and combination of BLV therapy requires further studies, especially with regard to the long-term outcome of treatment.

Treatment endpoints and the optimal treatment duration for BLV have not been defined yet. Indeed, to date, it is unclear whether BLV will be a long-term or a finite treatment in order to maximize the patient’s benefit. We report three cases that were treated with BLV (2 mg/day) plus TDF for longer than 48 weeks. Longer treatment resulted in a continuous virologic response in two out of the three patients followed for more than 48 weeks, with almost normalized ALT values at the end of observation. However, one patient showed a breakthrough of HDV RNA after an initial complete suppression. The patient confirmed that he was adhering to the treatment, which is plausible, as the increase in serum bile acids persisted. Moreover, serum ALT remained within normal ranges, suggesting the absence of active hepatitis despite a rebound of viral replication. Genotyping of HDV confirmed the persistence of HDV-1 and, thus, the reappearance of the initial virus strain. A similar observation was reported from a cohort in Berlin [25]. However, in contrast to the case in Berlin, the rebound in our patient occurred later in the course of treatment (40 weeks vs. 8 weeks in Berlin) and after a longer time of complete suppression of HDV RNA (28 weeks vs. 4 weeks in Berlin). A potential pathophysiological explanation for the viral rebound phenomenon might involve NTCP-independent cell-to-cell spread of HDV [29]. However, the concept remains speculative and requires further investigation.

As a potential factor of influence on the virologic response to treatment, we observed that the median HDV viral load at baseline was lower in virologic responders (patients who showed ≥2 log reduction in HDV RNA) compared to virologic non-responders (3.4 × 10^5^ vs. 9.8 × 10^6^ GEQ/mL). A similar correlation was previously reported for the treatment with PEG-IFNα [12,13,26]. Therefore, especially in patients with a higher viral load at baseline, a longer duration of treatment, higher doses of BLV, or with regard to the synergistic antiviral mechanisms [30], a combination of BLV with PEG-IFNα might be required to increase antiviral efficacy.

None of our patients showed HBsAg reduction or HBsAg loss, the only reliable endpoint indicating cure of HDV [31]. As the lower limit of detection of our HDV RNA assay was by far higher than the infectivity threshold of HDV in HBsAg carriers [15], residual HDV may reactivate any time after the cessation of treatment, even in complete virologic responders. Therefore, a relapse of HDV RNA in the case of cessation of treatment would be highly likely in our patients, in line with the high relapse rate of 93–97% in the MYR202 study after the end of therapy, and argues for an indefinite treatment duration of BLV in combination with TDF.

To conclude, we confirm under real-world conditions that the combination of BLV at the approved dose of 2 mg/day in combination with TDF is a safe, well-tolerated, and effective therapeutic option in patients with CHD for 24 weeks and beyond. Interestingly, viral breakthrough despite a biochemical response and compliance to BLV therapy can be observed in single cases. This phenomenon is currently poorly understood and requires further investigation. 

## 4. Patients and Methods

### 4.1. Patients

Patients with CHD who received treatment with BVL and TDF at the outpatient clinic of the University Hospital Leipzig for a minimum of 24 weeks from May 2020 to February 2022 were included. Clinical baseline characteristics before treatment were extracted from patient health records. All patients received treatment with BLV (2 mg/day) subcutaneously in combination with TDF (245 mg/day) per os. HBV DNA, HDV RNA, HBsAg, serum bile acids, ALT and the adverse effects of treatment were monitored at regular follow-up visits during the treatment period. 

### 4.2. Ethics Statement

The study was conducted according to the guidelines of the Declaration of Helsinki and approved by the Ethics Committee of the University of Leipzig (vote numbers 112/18-ek and 249/19-ek). Informed consent was obtained from all subjects involved in the study.

### 4.3. Molecular Diagnostic Assays

HDV RNA and HBV DNA were quantified by real-time polymerase chain reaction (PCR)-based assays (HDV: assay was performed as described before [32], lower limit of detection (LLD) 150 GEQ/mL; HBV: HBV Alinity m, Abbott Molecular, LLD 10 IU/mL), and HBsAg was quantified by the ARCHITECT assay (Abbott Molecular, sensitivity 0.05 IU/mL). HBV and HDV genotyping and the genotyping of *SLC10A1* for the SNPs rs6174593, rs2296651, rs72547507 and rs72547506 were performed as described in the Supplementary Material.

### 4.4. Definition of Response

Response to treatment was defined analogously to the criteria in the MYR202 trial [11]. Thus, a virologic response was defined as a ≥2 log reduction in HDV RNA or the suppression of HDV RNA below the lower limit of detection at week 24 of treatment. A biochemical response was defined as the normalisation of elevated serum ALT at week 24 of treatment. A combined treatment response was defined as a ≥2 log decline or suppression of HDV RNA in combination with the normalisation of elevated ALT levels at week 24 of treatment.

## Figures and Tables

**Table 1:** Baseline characteristics of the patient cohort. ALT, alanine aminotransferase; AST, aspartate aminotransferase; Geq, genome equivalents; GGT, gamma-glutamyl transferase; HBeAg, hepatitis B envelope antigen; HBV, hepatitis B virus; HDV, hepatitis D virus; LLN, lower limit of normal; n.a., not available; n.d., could not be determined due to low HBV DNA levels; PEG-IFNα, pegylated interferon alpha; TDF, tenofovir disoproxil fumarate; ULN, upper limit of normal.

	Patient 1	Patient 2	Patient 3	Patient 4	Patient 5	Patient 6	Patient 7
Gender, age (years)	Female, 34	Male, 59	Male, 44	Male, 33	Male, 44	Female, 39	Female, 35
Country of origin	Armenia	Russia	Russia	Romania	Albania	Uzbekistan	Moldova
Previous antiviral treatment	TDF, PEG-IFNα	TDF, PEG-IFNα	TDF, PEG-IFNα	None	TDF	None	None
HBV genotype	n.d.	HBV-D1	HBV-D2	n.d.	n.d.	n.d.	HBV-D1
HBV DNA (IU/mL)	33	<10	11	14	27	35	600
HBeAg	Negative	Negative	Negative	Negative	Negative	Negative	Negative
Anti-HBe	Positive	Positive	Positive	Positive	Negative	Positive	Positive
HDV genotype	HDV-1	HDV-1	HDV-1	HDV-1	HDV-1	HDV-1	HDV-1
HDV RNA (Geq/mL)	3220	2600	136,000,000	19,000,000	15,700,000	3,900,000	340,000
Cirrhosis (Child–Pugh score)	Yes (A)	Yes (A)	Yes (A)	No	No	Yes (A)	No
Liver stiffness (kPa)	21.1	21.5	13.9	8.4	14.2	19.9	n.a.
Hepatocellular carcinoma	No	Yes	No	No	No	No	No
ALT (IU/L) ULN ♀ < 35, ♂ < 45	28.8	71.4	115.8	150	303.6	127.8	45
AST (IU/L) ULN ♀ < 31, ♂ < 35	61.8	37.8	68.4	73.8	133.2	101.4	39
GGT (IU/L) ULN ♀ < 38, ♂ < 55	70.8	174	204	34.2	36	30.6	28.2
Total bilirubin (mg/dL) ULN ♀/♂ < 1	1	0.38	0.96	0.45	0.28	0.71	0.15
Albumin (g/L) LLN ♀/♂ < 35	42.2	45.1	46.9	48.1	46.6	48.9	40
Platelets (1000/µL) LLN ♀/♂ < 150	41	120	160	204	145	158	376

**Table 2:** Genotyping of *SLC10A1* for disease-modifying polymorphisms. *SLC10A1*, solute carrier family 10 member 1 (coding gene for NTCP).

*SLC10A1* Polymorphism	Genotype
rs61745930 T > A	TT (wildtype) in all patients
rs2296651 C > G	CC (wildtype) in all patients
rs71547507 T > A	TT (wildtype) in all patients
rs72547506 A > T	AA (wildtype) in all patients

## Data Availability

The data presented in this study are available on request from the corresponding author.

## Supplementary Materials

The following supporting information can be downloaded at: https://www.mdpi.com/article/10.3390/pathogens11050517/s1, Table S1: Sequence of primers and probes for melting curve analysis and sequencing.
