# Supplementary material for: Efficacy and Safety of Bulevirtide plus Tenofovir Disoproxil Fumarate in Real-World Patients with Chronic Hepatitis B and D Co-Infection
Source: Pathogens. 2022 Apr 27;11(5):517. doi: 10.3390/pathogens11050517 (PMC9143982; doi:10.3390/pathogens11050517)
Supplement: Supplementary file 1 [file pathogens-11-00517-s001.zip › pathogens-1693937-supplementary.pdf]

Supplementary Methods

HBV and HDV genotyping

For HBV genotyping, the primers HBV11F (5′ACTCGTGGTGGACTTCTC3′) and HBV11R (5′GGGTTGCGTCAGCAAACAC3′) were used to amplify a fragment of approximately 900 bp of the overlapping surface/polymerase region.

PCR was conducted on a block-cycler system in a final volume of 50 μL with a final concentration of 1.5 mM MgCl2, 1-fold buffer, 10 pmol of each primer (Metabion, Martinsried, Germany), 250 mM dNTP's (Roche, Mannheim, Germany), 2 U Platinum TaqPolymerase (Invitrogen, Karlsruhe, Germany) and 10 μL of template DNA. The cycling conditions were 95 °C for 2 min, followed by 45 cycles at 95 °C for 30 sec, 58 °C for 30 sec and 72 °C for 2 min. For HDV genotyping, the amplification products from the quantitative real-time PCR were used for sequencing.

The HBV and HDV PCR products were gel-purified using the Wizard SV Gel and PCR Clean-Up System (Promega, Mannheim, Germany). The DyeTerminator v.1. cycle Sequencing Kit (Applied Biosystems, CA, USA) was used to sequence the fragments in both directions on an Applied Biosystems 3500 Genetic Analyzer (Applied Biosystems, CA, USA).

Genotyping of *SLC10A1*

The genomic DNA was extracted from whole blood samples with an extraction kit from QIAGEN (Hilden, Germany). Genotyping of the *SLC10A1* SNPs rs6174593, rs2296651, rs72547507 and rs72547506 was performed with a Light Cycler® 480 System (Roche, Basel, Switzerland). Polymerase chain reaction (PCR) amplification and melting curve analysis was carried out in a total volume of 10 μL containing 5.0 μL of Light Cycler 480 Probes Master with 6.4 mM $MgCl_2$, 3.6 μL PCR-grade H_2O, each primer (0.5 mM), a 0.1 mM sensor probe and a 0.1 mM anchor probe. The PCR conditions were: initial denaturation at 95 °C for 12 min; followed by 45 cycles of denaturation at 95 °C for 20 sec; annealing at 60 °C for 40 sec and extension at

72 °C for 1.5 min. The melting curves were detected after denaturation at 95 °C for 5 sec, holding the sample at 40 °C for 20 sec and then heating the sample to 80 °C with a ramp rate of 0.14 °C/sec and continuous fluorescence acquisition. The primer and probe sequences are shown in **Supplementary Table S1**. Sequencing was performed with BigDye Terminator and a capillary sequencer from Applied Biosystems (Darmstadt, Germany).

Supplementary Table S1. Sequence of primers and probes for melting curve analysis and sequencing. FL, Fluorescein; LC, Light Cycler; Ph, phosphate; SNP, single-nucleotide polymorphism.

***SLC10A1* SNPs**	**Primer/Probes**	**Sequence (5′-3′)**
rs61745930	sense	ACCTCTGTTCCTCTCTATC
	antisense	TGAGCTGAGAATGTGCTAC
	sensor	ACACCACTCTTGACTGCC-FL
	anchor	LC640-CCTCCTCCCTGATGCCTTTTA-Ph
rs2296651<br>rs71547507<br>rs72547506	sense	TCCAGTTCCCTCTGAGTG
	antisense	CTACCTGGTTCTTAGTGAC
rs2296651	sensor	CAACTCTGTTCCACCATCC-FL
	anchor	LC640-CAATGTGGCCTTTCCACCTGA-Ph
rs71547507	sensor	ACCTGAAGTCACTGGACC-FL
	anchor	LC640-CTTTTCTTCTTTCCCCTC CTCT-Ph
rs72547506	sensor	CTCCCGAGGGTGAGTAC-FL
	anchor	LC640-GAAATTATCCCCACTTCAAGTTCTG-Ph
